# Mediating effect of self-efficacy on the relationship between Big Five personality and medication adherence in Chinese hypertensive patients: a national cross-sectional study

**DOI:** 10.3389/fpsyg.2024.1442031

**Published:** 2024-10-30

**Authors:** Mingliang Du, Yibo Wu, Boran Wang, Miao Jiang, Jiangyun Chen, Hui Hui

**Affiliations:** ^1^Department of Coronary Heart Disease, Central Hospital of Dalian University of Technology (Dalian Municipal Central Hospital), Dalian, China; ^2^School of Public Health, Peking University, Beijing, China; ^3^Medical Record Room, Central Hospital of Dalian University of Technology (Dalian Municipal Central Hospital), Dalian, China; ^4^School of Health Management, Southern Medical University, Guangzhou, China

**Keywords:** hypertension, personality, self-efficacy, medication adherence, mediating effect

## Abstract

**Objective:**

The study aims to evaluate personality characteristics, self-efficacy, and medication adherence in Chinese patients with hypertension, while also investigating how self-efficacy mediates the relationship between personality traits and medication adherence.

**Methods:**

This investigation included 787 Chinese patients diagnosed with hypertension, as reported in the “2021 China Family Health Index Survey Report.” The study employed several assessment tools such as a General Information Questionnaire, the Brief Big Five Inventory (BFI-10), the New General Self-Efficacy Scale (NGSES), and the Medication Adherence Rating Scale (MARS). Statistical analyses encompassed the Mann–Whitney U test, chi-square test, multiple logistic regression, Spearman’s rank correlation, standardized regression coefficients, and Bootstrap techniques.

**Results:**

(1) Individuals with debt, who also possess high levels of self-efficacy, tend to adhere more consistently to their medication regimens. (2) An analysis of personality traits indicated that Extroversion,Conscientiousness and Openness positively influences self-efficacy,while Agreeableness and Neuroticism negatively impacts it. (3) Self-efficacy plays a mediating role in the indirect relationship between personality traits such as Extroversion and neuroticism and medication adherence.

**Conclusion:**

In the context of Chinese adults, both self-efficacy and Extroversion positively influence medication adherence, whereas high levels of neuroticism adversely impact it. Furthermore, self-efficacy serves as a mediating factor in the linkage between personality traits and medication adherence.

## 1 Introduction

The 2019 Global Burden of Disease Report revealed that the top three diseases contributing to the global disease burden were ischemic heart disease, diabetes, and stroke. Both stroke and ischemic heart disease are closely linked to hypertension, making the proactive prevention and control of hypertension the core strategy in mitigating the prevalence of cardiovascular and cerebrovascular diseases in China. Research indicates that inadequate medication adherence correlates with adverse health outcomes and increased healthcare expenditures. In China, the rate of adherence to medication among individuals diagnosed with hypertension stands at a mere 42.5%. This figure is notably lower compared to the average adherence rate of 52.0% observed across other Asian nations. The guidelines issued by the European Society of Hypertension and the European Society of Cardiology (ESH/ESC) in 2018 highlight the importance of assessing adherence in patients, especially in cases where optimal blood pressure management has not been attained (Mahmood et al., 2021; Williams et al., 2018). This non-adherence can have a detrimental influence on patient health status and may generate other complications, such as a reduced quality of life, greater risk of hospitalization, and increased health care costs (Gauterio Abreu et al., 2018). Adherence to medications is associated with several circumstances related to the patient (cognitive function, impaired sight, lack of understanding, inability to administer different medications), with the pathology, with cultural health beliefs, with habits, and with quality of life, as well as with the relationship with the healthcare team. It is important to point out that behavioral patterns can also directly influence patients’ pharmacological adherence, as they are related to their perception and understanding of their own health status and drug and non-drug therapies (Gauterio Abreu et al., 2018).

Personality traits are enduring behavioral patterns that tend to be stable throughout life. They set the standards of behavior and interpersonal relationships. Therefore, personality traits can also influence adherence behavior. According to the Five Factors Model (Big Five), people’s personality traits can be defined as predispositions to behave in a certain way and can be grouped into five major dimensions: Neuroticism, Extraversion, Openness to Experience, Agreeableness, and Conscientiousness (Linkievicz et al., 2022). However personality characteristics are one of the factors influencing medication adherence, yet they tend to stabilize in later adulthood (O'Shea et al., 2017). Consequently, some scholars have investigated self-efficacy as a mediating factor. Self-efficacy was initially introduced by Bandura (1977). It pertains to an individual’s conscious organization and implementation of specific behaviors, with confidence in their ability to achieve specific goals. It is primarily achieved through self-management and regulation of one’s behavior, thereby impacting the effectiveness of outcomes. The self-efficacy theory is a core viewpoint of cognitive theory in modern sociology, affecting survival status, goal setting, thinking patterns, behavioral motivations, and coping mechanisms with difficulties and setbacks. In the management of hypertension, self-efficacy as a mediator may offer a new perspective. To date, the mediating effect of self-efficacy on personality and medication adherence has not been confirmed, and its universality and practicality in the Chinese population with hypertension still require validation.

Few studies have investigated the role of self-efficacy in big five personality and medication adherence among patients with hypertensive. Exploring the role of self-efficacy in the relationship between personality traits and medication adherence is helpful to develop effective interventions to improve medication adherence of patients with hypertensive. Thus, this study utilized the 2021 China Family Health Index Survey Report to perform a comprehensive cross-sectional study, encompassing 11,031 participants across China. It included 787 adult hypertensive patients in its analysis. This research was designed to evaluate personality characteristics, self-efficacy, and the extent of medication compliance in Chinese hypertensive patients, investigate the relationships among these factors, and formulate a theoretical model that demonstrates how self-efficacy serves as a mediator in the nexus between personality traits and adherence to prescribed medications. The insights generated from this study are expected to provide new strategies for hypertension management.

## 2 Objects and methods

### 2.1 Objects

A structured survey was implemented across Mainland China from July 10 to September 15, 2021, employing a multi-stage sampling strategy. The investigation spanned the capital cities of twenty-three provinces, five autonomous regions, and four municipalities that are directly governed by the central government. Furthermore, for each province and autonomous region, a selection of two to six additional non-capital cities was made using the random number table method. This method ensured a wide and diverse representation, incorporating a total of 120 cities in the study. To align the sample demographics with the national statistics, quota sampling was utilized, adhering to the gender, age, and urban–rural distributions as documented in the 2021 7th National Population Census (Yuqi et al., 2024; Yibo et al., 2024). All participants provided informed consent, and the Questionnaire Star platform was used to administer questionnaires directly and personally. Respondents could also complete the questionnaire online via a provided link. For respondents who had the cognitive ability but lacked the physical capacity to complete the questionnaire, investigators conducted one-to-one interviews and recorded their responses. The research subjects were Chinese adults, with inclusion criteria as follows: ① Age over 18 years; ② Citizenship of the People’s Republic of China; ③ Permanent residency in China (traveling abroad for no more than 1 month annually); ④ Willingness to participate and complete an informed consent form; ⑤ Ability to independently complete online questionnaires or do so with assistance from investigators; ⑥ Comprehension of each item on the questionnaire was ensured. The exclusion criteria included: ① Individuals diagnosed with cognitive impairments or mental health disorders; ② Currently participating in similar studies; ③ Expressing a lack of willingness to cooperate. The ethical review and approval for this study were granted by the Ethics Committee of Jinan University, under the reference number JNUKY-2021-018.

In this survey, 11,709 questionnaires were issued, of which 11,031 were returned and deemed valid, representing a response rate of 90.34%. The sample included 9,966 adults aged 18 and above, with 4,591 males (46.1%) and 5,375 females (53.9%), and an average age of 38.02 years. Among them, 787 adult hypertensive patients were identified based on the questionnaire responses, comprising 447 males (56.8%) and 340 females (43.2%).

## 3 Methods

### 3.1 Research tools

①The General Demographic Data Questionnaire, a custom-designed instrument, encompasses various parameters including gender, age, body mass index (BMI), histories of smoking and alcohol consumption, marital and employment status, existing debts, average monthly income per family member, medical insurance type, and daily medications. ②The Brief Big Five Inventory (BFI-10) (Carciofo et al., 2016), crafted by Rammstedt and John (2007), simplifies the original personality scale. Operating under the Big Five personality framework, it evaluates five traits: Extroversion, agreeableness, conscientiousness, neuroticism, and openness through 10 items. Each trait is measured by two specific items, employing a 5-point Likert scale (ranging from 1 to 5 points), with items 2, 6, 8, 9, and 10 receiving positive scores and items 1, 3, 4, 5, and 7 scored in reverse. Higher aggregate scores suggest stronger traits, and the BFI-10 is noted for its robust reliability and validity (Carciofo et al., 2016). ③ The New General Self-Efficacy Scale (NGSES), established by Chen G, Gully M, and Eden D in 2001, consists of eight items within a singular dimension. It utilizes a 5-point Likert scale, achieving a maximum score of 40 points. Elevated scores reflect enhanced self-efficacy, with documented internal consistency between 0.85 and 0.90 and test–retest reliability indices ranging from 0.62 to 0.65 (Chen et al., 2001; Henson, 2001). ④ Medication Adherence Rating Scale (MARS): Medication adherence is assessed using the Chronic Disease Patient Medication Compliance Scale (MARS) (Thompson et al., 2000), consisting of 10 items, and is a simplified version of the Medication Compliance Questionnaire (MAQ) (Morisky et al., 2008). It evaluates patients’ adherence to medication, their attitudes toward medication, and overall disease management over the past week. Evidence suggests strong internal consistency and dependability of the scale, indicated by a Cronbach’s alpha value of 0.75. Scores for medication adherence range from 0 to 10 points. With the data exhibiting a normal distribution, a median score of 5 serves as the threshold to divide patients into two categories: those with high adherence, scoring above 5 points, and those with low adherence, scoring 5 points or less.

### 3.2 Quality control

To ensure the quality of the questionnaire, a one-to-one, face-to-face approach was adopted to guarantee the accuracy and authenticity of the information. The screening criteria included: ① Questionnaires with a response time of less than 240 s; ② Questionnaires with inconsistent logic checks; ③ Questionnaires with incomplete information; ④ Repeatedly submitted questionnaires; ⑤ Questionnaires where all selected options were identical or showed a regular pattern.

### 3.3 Statistical analysis

Utilizing software SPSS version 25.0, the analysis was conducted on data adhering to a normal distribution, employing paired *t*-tests to elucidate the outcomes, which were expressed as the mean ± standard deviation, whereas M (P25, P75) denoted the results of parallel Mann–Whitney U tests for data not following a normal distribution. Frequencies were reported as cases (%) and analyzed via the chi-square test. The factors influencing medication adherence among hypertensive patients were explored through multiple logistic regression analysis. To explore the relationships among personality traits, self-efficacy, and medication adherence, Spearman correlation analysis and standardized regression techniques were applied. Demographic variables were controlled using the PROCESS plugin, and the Bootstrap method was utilized to develop a mediation model linking personality, self-efficacy, and depression. If the direct effect was not significant, the effect of the mediating variable was a full mediating effect. If the direct effect was significant, the effect of the mediating variable was a partial mediating effect. a*b was used to calculate the mediation effect value, and a*b/c was used to represent the ratio of the mediation effect to the total effect. PROCESS macro with Model 4 for SPSS was used to test for mediation effects using 5,000 bootstrap samples. Mediation effects were considered to be significant if the 95% bootstrap confidence interval did not include zero. In addition, sociodemographic and clinical characteristics were included in the model as control variables. *p* < 0.05 was considered statistically significant.

## 4 Results

### 4.1 Demographic characteristics of patients in two groups

The male proportion, age, BMI, smoking history, alcohol consumption history, occupational status, marital status, type of medical insurance, family income, and type of medication were not statistically significant between patient groups with varying levels of medication adherence (all *p* > 0.05). However, a notably higher proportion of individuals with high medication adherence was observed to have debts (*p* < 0.05). For additional details, please consult Table 1.

**Table 1:** Comparison of demographic characteristics between two groups of patients [*n* (%)].

	Low medication adherence (*n* = 495)	High medication adherence (*n* = 292)	Z/χ^2^ value	*p* value
Gender (male)	281 (56.8)	166 (56.8)	0.00	0.982
Age, year				
18–35	14 (2.8)	6 (2.1)	0.564	0.754
35–55	189 (38.2)	116 (39.7)		
>55	292 (60.0)	170 (58.2)		
BMI (Weight/Height^2^)	23.4 (21.3,25.5)	23.7 (21.1,26.0)	−0.700	0.484
Smoking history	193 (39.0)	114 (39.0)	0.00	0.989
Alcohol consumption history	207 (41.8)	142 (48.6)	3.45	0.063
Occupational status				
Unemployed	159 (32.1)	98 (33.6)	0.244	0.894
Retired	187 (37.8)	106 (36.3)		
Employed	149 (30.1)	88 (30.1)		
Marital status				
Unmarried	10 (2.0)	6 (2.0%)	1.414	0.493
Married	420 (84.8)	256 (87.7)		
Divorced/Widowed	65 (13.1)	30 (10.3)		
Medical insurance type				
Self-funded	52(10.5)	36 (12.3)	0.634	0.728
Public-funded	8(1.6)	5 (1.7)		
Medical insurance	435(87.9)	251 (86.0)		
In debt	118(23.8)	110 (37.7)	17.08	<0.001
*Per capita* household income (Yuan)				
<3,000	147(29.7)	84 (28.8)	0.105	0.949
3,000–6,000	215(43.4)	127 (43.5)		
≥6,000	133(26.9)	81 (27.7)		
Medication type (Types)				
1	216(43.6)	124 (42.5)	0.109	0.947
2	185(37.4)	112 (38.4)		
≥3	94(19.0)	56 (19.2)		

### 4.2 Refinement of multivariate logistic regression analysis on medication adherence in hypertensive patients

A multivariate logistic regression was conducted to assess factors influencing medication adherence, defined as binary outcomes (low adherence = 0, high adherence = 1). Independent variables included demographic and health-related factors such as gender, age, BMI, smoking history, and alcohol consumption history, as well as socio-economic and psychological variables such as occupational status, marital status, type of medical insurance, current financial debts, income per household member, number of prescribed medications, and levels of self-efficacy. The analysis revealed that financial obligations and self-efficacy levels are significant determinants of medication adherence. Specifically, higher levels of debt and self-efficacy were associated with increased adherence, as detailed in Table 2, with statistical significance noted (*p* < 0.05).

**Table 2:** Regression analysis of factors influencing medication adherence.

Variable	*B*	Standard deviation	Wald	Degree of freedom	Significance	Exp (B)	Exp (B) 95%	
							Upper limit	Lower limit
Gender	−0.147	0.193	0.583	1	0.445	0.863	0.591	1.259
Age, year (Reference group = 18–35)			0.253	2	0.881			
Age (35–55)	−0.033	0.451	0.005	1	0.942	0.968	0.400	2.343
Age (= > 55)	−0.136	0.470	0.084	1	0.771	0.872	0.348	2.190
BMI	0.001	0.023	0.004	1	0.953	1.001	0.958	1.047
Smoking history	−0.021	0.190	0.012	1	0.912	0.979	0.674	1.422
Alcohol consumption history	0.208	0.182	1.307	1	0.253	1.232	0.862	1.760
Occupational status (Reference group = Unemployed)			2.151	2	0.341			
Occupational status (Retired)	−0.309	0.215	2.078	1	0.149	0.734	0.482	1.118
Occupational status (Employed)	−0.152	0.224	0.459	1	0.498	0.859	0.554	1.333
Marital status (Reference group = Unmarried)			2.005	2	0.367			
Marital status (Married)	−0.600	0.464	1.669	1	0.196	0.549	0.221	1.363
Marital status (Divorced/Widowed)	−0.743	0.528	1.985	1	0.159	0.476	0.169	1.337
Medical insurance type (Reference group = Self-funded)			0.111	2	0.946			
Medical insurance type (Public-funded)	−0.152	0.652	0.054	1	0.816	0.859	0.239	3.086
Medical insurance type (Medical insurance)	0.036	0.251	0.021	1	0.884	1.037	0.634	1.696
In debt	0.479	0.176	7.435	1	0.006	1.615	1.144	2.278
*Per capita* household income (Reference group = <3,000)			0.038	2	0.981			
*Per capita* household income (3,000–6,000)	−0.003	0.189	0.000	1	0.985	0.997	0.688	1.444
*Per capita* household income (≥6,000)	−0.037	0.219	0.029	1	0.864	0.963	0.627	1.479
Medication type (Reference group = 1)			0.372	2	0.830			
Medication type (2 types)	0.100	0.172	0.339	1	0.560	1.105	0.790	1.547
Medication type (3 types)	0.084	0.214	0.155	1	0.694	1.088	0.715	1.655
Self-efficacy	0.038	0.016	5.270	1	0.022	1.039	1.006	1.073

### 4.3 The relationship between Big Five personality traits and medication adherence: the mediating role of self-efficacy

Step 1 encompassed executing a multiple linear regression analysis, where the focal point was to evaluate how the Big Five personality traits could potentially predict medication adherence. This regression assessment discerned that these personality dimensions failed to display any significant predictive power concerning adherence behaviors (*p* > 0.05). In Step 2, another regression was carried out, this time with self-efficacy as the dependent variable and the Big Five traits as predictors. The analysis identified that Extroversion positively influenced self-efficacy (*β* = 0.444, *p* < 0.001), while agreeableness negatively affected it (*β* = −0.487, *p* < 0.001). Conscientiousness was found to enhance self-efficacy significantly (*β* = 0.959, p < 0.001), neuroticism decreased it (*β* = −0.835, *p* < 0.001), and openness also had a positive effect (*β* = 0.523, *p* < 0.001). Step 3 utilized a regression model with medication adherence as the outcome and incorporated both the Big Five traits and self-efficacy as predictors. The results indicated that self-efficacy significantly improved medication adherence (*p* < 0.05), whereas Extroversion, agreeableness, and openness did not significantly impact adherence (*p* > 0.05). For more details, please see Table 3.

**Table 3:** The relationship between Big Five personality traits and medication adherence: the mediating role of self-efficacy.

Step	Dependent variable	Independent variable	*β*	SE	*t*	*R*^2^	*R*^2^ variation
Step 1	Medication adherence	Extroversion	−0.074	0.056	−1.335	0.002	0.002
		Agreeableness	−0.015	0.063	−0.233	<0.001	<0.001
		Conscientiousness	−0.026	0.052	−0.503	<0.001	<0.001
		Neuroticism	0.059	0.059	1.001	0.001	0.001
		Openness	0.042	0.055	0.754	0.001	0.001
Step 2	Self-efficacy	Extroversion	0.444	0.120	3.699***	0.017	0.017
		Agreeableness	−0.487	0.137	−3.558***	0.016	0.016
		Conscientiousness	0.959	0.109	8.825***	0.090	0.090
		Neuroticism	−0.835	0.124	−6.724***	0.054	0.054
		Openness	0.523	0.119	4.394***	0.024	0.024
Step3	Medication adherence	Extroversion	−0.088	0.056	−1.583	0.007	0.007
		Self-efficacy	0.032	0.016	1.967		
		Agreeableness	−0.001	0.064	−0.010	0.004	0.004
		Self-efficacy	0.029	0.016	1.757		
		Conscientiousness	−0.059	0.055	−1.087	0.005	0.005
		Self-efficacy	0.035	0.017	2.018*		
		Neuroticism	0.088	0.060	1.458	0.007	0.007
		Self-efficacy	0.035	0.017	2.066*		
		Openness	0.027	0.056	0.486	0.004	0.004
		Self-efficacy	0.028	0.017	1.676		

^*^P < 0.05; ^***^p < 0.001.

### 4.4 The mediating effect test of Big Five personality-self-efficacy-medication adherence

**Figure 1:**
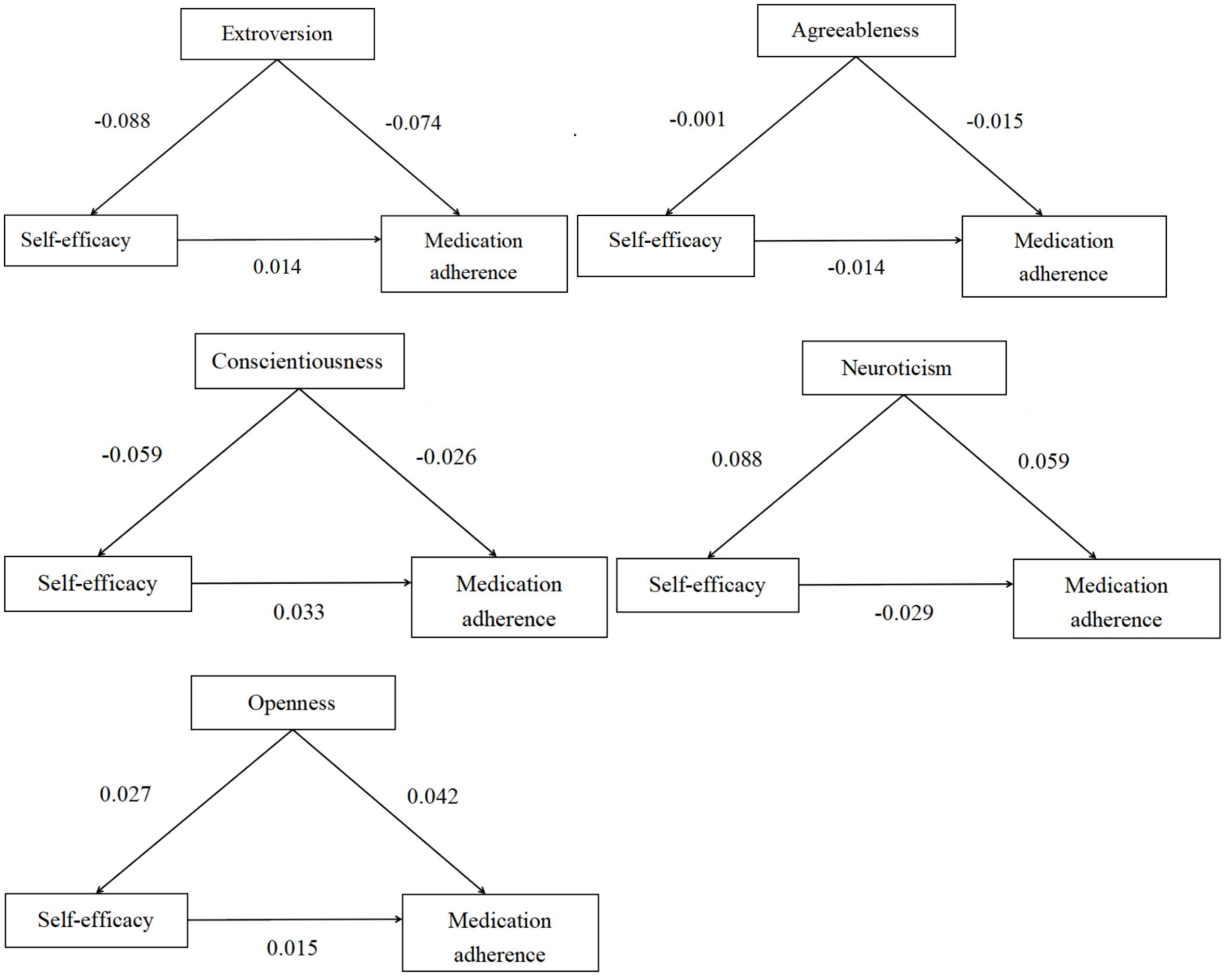
Schematic diagram of the mediating effect analysis of self-efficacy between Big Five personality traits and medication adherence.

The mediating effect of the indirect pathway “Big Five personality-self-efficacy-medication adherence” was tested using the Process plugin model 4 in SPSS. The Bootstrap sample size was 5,000, and the 95% confidence interval (CI) did not include 0, indicating a significant mediating effect. It was observed that, except for the indirect effects of “Extroversion-self-efficacy-medication adherence” and “neuroticism-self-efficacy-medication adherence,” which did not include 0, the mediation effects were significant. However, the indirect effects of the other three pathways all included 0, indicating non-significance of the mediating effect. Please refer to Table 4 and Figure 1 for further details.

**Table 4:** The mediating effect test of Big Five personality-self-efficacy-medication adherence.

Item	Effect relationship	Effect size	Boot *SE*	95% CI	
				Upper limit	Lower limit
Extroversion-self-efficacy-medication adherence	Total effect	−0.074	0.056	−0.183	0.035
	Direct effect	−0.088	0.056	−0.198	0.021
	Indirect effect	0.014	0.009	<0.001	0.038
Agreeableness-self-efficacy-medication adherence	Total effect	−0.015	0.063	−0.139	0.109
	Direct effect	−0.001	0.064	−0.126	0.124
	Indirect effect	−0.014	0.010	−0.039	0.001
Conscientiousness-self-efficacy-medication adherence	Total effect	−0.026	0.052	−0.129	0.076
	Direct effect	−0.059	0.055	−0.167	0.048
	Indirect effect	0.033	0.019	−0.001	0.073
Neuroticism-self-efficacy-medication adherence	Total effect	0.059	0.059	−0.056	0.174
	Direct effect	0.088	0.060	−0.030	0.206
	Indirect effect	−0.029	0.016	−0.064	−0.001
Openness-self-efficacy-medication adherence	Total effect	0.042	0.055	−0.067	0.150
	Direct effect	0.027	0.056	−0.083	0.137
	Indirect effect	0.015	0.010	−0.002	0.039

## 5 Discussion

In comparison with previous studies, the present study has certain advantages in terms of scale selection. First, the NGSES has better internal consistency (0.85 to 0.90) and stability than other self-efficacy scales such as the Personal Efficacy Scale, the Generalized Self Efficacy Scale, and the General Perceived Self-Efficacy Scale (Chen et al., 2004; Chen et al., 2001). The internal consistency coefficients of NGSES in the present study ranged from 0.92 to 0.96.Moreover, the NGSES has no specific requirements for respondents to complete the questionnaire, making it suitable for large-scale surveys.This study used MARS, which indicates problematic behavior through MAQ questions and attitudes based on DAI items, making it more helpful for measuring medication adherence in individual patients. The selection of suitable scales resulted in comprehensive and accurate measurements of self-efficacy, personality traits and medication adherence among patients with chronic diseases.

Medication adherence is influenced by various factors. Low adherence not only reduces the control rate of hypertension but also escalates medical costs. Therefore, identifying these factors is paramount. Self-efficacy, reflecting one’s belief in their ability to achieve desired outcomes through their actions, emerged as a significant factor. The study indicated that higher self-efficacy correlated with increased medication adherence (OR = 1.039, 95% CI = 1.006–1.073, *p* = 0.022). A study focusing on patients with coronary heart disease revealed that 61.8% demonstrated a high level of medication adherence, which correlated strongly with elevated self-efficacy levels (Mobini et al., 2023). In their investigation into the determinants of medication adherence among individuals with cardiovascular diseases, Al-Ganmi et al. (2019) found that self-efficacy significantly improved the likelihood of adhering to prescribed medication schedules. Consistent with these findings, Shen et al. (2020) reported a significant positive association between self-efficacy and medication adherence, highlighting self-efficacy as a key independent predictor in this regard. This might be because patients with higher self-efficacy have more confidence in their ability to adhere to medication regimens. Enhancing self-efficacy could be a viable approach to improving medication adherence. A randomized controlled trial demonstrated that implementing intervention strategies based on the health behavior process approach markedly enhanced self-efficacy and, consequently, significantly increased medication adherence among patients with type 2 diabetes (Ranjbaran et al., 2022).

This study found no statistically significant difference in personality traits among patients with varying levels of medication adherence (*p* > 0.05). Studies within the community hypertension population indicate that there is a negative correlation between neuroticism and medication adherence, whereas both conscientiousness and Extroversion show positive correlations with adherence. In other words, patients with higher levels of conscientiousness and Extroversion tended to have better medication adherence, while those with higher neuroticism exhibited poorer adherence, suggesting a link between personality traits and medication adherence (Fanfan et al., 2016). Another study on glaucoma patients showed a negative correlation (*p* < 0.001) between neuroticism and medication adherence (Abu et al., 2023).

However, finally, the mediating role of self-efficacy in the relationship between personality traits and medication adherence was an important finding in our study.By investigating the mediating role of self-efficacy in the relationship between social support and medication adherence in patients with previous failure in America, Maeda et al. (2013) after controlling demographic variables, such as age, gender, and education, emphasized the mediating role of self-efficacy. Also, Xie et al. (2020) reported the same results among patients with coexisting type 2. Self-efficacy might be a key target for implementing interventions to improve self-care behaviors in these patients. Our findings suggest that Extroversion and neuroticism can indirectly influence medication adherence through self-efficacy, which positively impacts adherence. Self-efficacy has a positive mediating effect between Extroversion and medication adherence, while it has a negative effect between neuroticism and medication adherence. Compared to personality traits, self-efficacy is more amenable to change and potentially less costly, emphasizing the importance of enhancing self-efficacy. Thus, future investigations may focus on identifying specific factors that affect self-efficacy and how these factors can improve self-efficacy to enhance patient medication adherence.

This study has some limitations. First, self-report instruments were used to measure medication adherence, which may have led to an overestimation of adherence. Future studies could use objective data as an indicator of medication adherence. Second, this was a cross-sectional study using a purposive sampling method, which did not allow us to infer causal relationships among personality traits, self-efficacy and medication adherence. Finally, this study was conducted in China, which limits the generalizability of the findings. Future multicenter studies are warranted. At the same time, the timing of the survey during the COVID-19 pandemic could have influenced the mental health of participants and may have introduced recall biases.

## Data Availability

The datasets presented in this study can be found in online repositories. The names of the repository/repositories and accession number(s) can be found in the article/supplementary material.
